# A pharmacokinetic study of radiprodil oral suspension in healthy adults comparing conventional venous blood sampling with two microsampling techniques

**DOI:** 10.1002/prp2.459

**Published:** 2019-01-28

**Authors:** David Sciberras, Christian Otoul, Françoise Lurquin, John Smeraglia, Aurélia Lappert, Steven De Bruyn, Jan Jaap van Lier

**Affiliations:** ^1^ UCB Biopharma SPRL Braine l'Alleud Belgium; ^2^ PRA Health Sciences – Early Development Services (PRA‐EDS) Groningen The Netherlands

**Keywords:** bioequivalence, drug safety, pharmacokinetics, phase 1

## Abstract

In this phase I, single‐center, open‐label study of ten heathy adults (18‐45 years; NCT02647697), the PK, safety, and tolerability profile of radiprodil oral suspension in healthy adults were assessed, as well as two PK microsampling techniques. All participants received a single 30 mg radiprodil dose (12 mL oral suspension). Blood was collected at various time points using conventional venous sampling (intravenous catheter or venepuncture), and Mitra™ and Aqua‐Cap™ Drummond microsampling (finger‐prick and blood taken from venous blood sample tubes). Geometric mean radiprodil plasma concentrations from conventional venous samples were above the lower limit of quantification up to 48 hours after administration of a single oral dose of radiprodil. Geometric mean AUC_inf_ and *C*_max_ were 2042 h ng mL ^−1^ and 89.4 ng mL ^−1^, respectively. Geometric mean *t*_&frac12;_ was 15.8 hour; median *t*_max_ was 4 hour (range: 3‐6 hour). Radiprodil exposure variables for Aqua‐Cap™ Drummond sampling were similar to the conventional venous‐derived data. Conversely, radiprodil exposure variables were lower with Mitra™ sampling compared with conventional venous sampling. The geometric mean ratio (90% confidence interval) for *C*_max_ of conventional venous versus Mitra™ and Aqua‐Cap™ Drummond sampling (finger‐prick blood) was 0.89 (0.85, 0.94) and 1.03 (0.97,1.08), respectively, and therefore within the conventional bioequivalence range (0.80‐1.25). Radiprodil oral suspension had an acceptable safety, tolerability, and palatability profile. The PK profile of radiprodil oral suspension was established in healthy adults, and was comparable when analyzed using conventional versus microsampling techniques. These results will support future radiprodil paediatric studies.

## 1 INTRODUCTION

Radiprodil (UCB3491) is a selective negative allosteric modulator of NR2B‐containing N‐methyl‐d‐aspartate (NMDA) receptors and is currently under development for the treatment of infantile spasms (IS). IS is a severe infantile seizure disorder, in which onset of seizures usually occurs within the first year of life, with a peak age of onset of 3‐5 months.^1^

An oral suspension formulation of radiprodil has been developed specifically for paediatric use. Before initiating any paediatric research, however, this first‐in‐human study for this formulation of radiprodil was carried out with the primary objective of evaluating the pharmacokinetics (PK) of radiprodil oral suspension in healthy adults. Secondary objectives were to evaluate safety and tolerability in this adult population. Exploratory objectives were to evaluate the palatability of radiprodil oral suspension and to compare conventional venous blood sampling with two microsampling techniques that sampled both finger‐prick blood and blood taken from the venous blood sample tubes.

Mitra™ volumetric absorbent microsamplers (VAMS) absorb a small sample of blood, which, after air‐drying, can be used for subsequent bioanalysis.^2^ Aqua‐Cap™ Drummond capillary tubes comprise a glass capillary blood sampling system for the analysis of small, exact volumes of plasma.^3^ Both these techniques have the potential to overcome some of the practical challenges of evaluating PK in infants, as the blood volume required is small and can be obtained without venepuncture. It is reasonable therefore, to compare the two techniques with conventional venous blood sampling prior to paediatric development research. This is the first study with this formulation of radiprodil in humans.

## 2 METHODS

### 2.1 Study design and participants

This was a phase I open‐label study in healthy adults (UP0027/NCT02647697, ClinicalTrials.gov) conducted at a single center in the first quarter of 2016. The study consisted of a 21‐day screening period, 3‐day treatment period, and end of study (EOS) visit 9‐14 days post dose.

Ten healthy male and female participants 18‐45 years of age with body mass indexes between 18.0 and 30.0 kg·m^−^² were enrolled. Participants entered the study center the afternoon before administration, remaining until 48 hours post administration. Each study participant received a single oral dose of radiprodil 30 mg in 12 mL suspension, via a syringe. Radiprodil was administered in the morning of Day 1 after an overnight fast of at least 12 hours.

Adverse events (AEs) were assessed throughout the study. Vital signs were measured predose, at several time points until 24 hours post dose, and at the EOS visit. A resting 12‐lead electrocardiogram (in supine position after 5 minutes rest) and physical examination were performed predose, 24 hours post dose, and at the EOS visit, as were measurements for hematology, biochemistry, and urinalysis parameters. Palatability of the radiprodil suspension was evaluated using three questions on taste and texture, asked immediately following dosing.

The study was conducted according to the International Conference on Harmonisation and the Declaration of Helsinki, and the study protocol was reviewed and approved by an independent medical ethics committee (Stichting Beoordeling Ethiek Biomedisch Onderzoek, the Netherlands; Central Committee on Research involving Human Subjects number NL55708.056.15). All participants provided their informed consent in writing prior to the start of study procedures.

### 2.2 Blood sampling

Blood samples (2 mL) for PK analysis were collected into EDTA tubes via intravenous catheter or direct venepuncture (conventional venous sampling) at the following time points: predose, and 0.5, 1, 1.5, 2, 2.5, 3, 3.5, 4, 6, 8, 12, 24, 36, and 48 hours post dose. Plasma for measurement of radiprodil was harvested by centrifugation of blood samples.

### 2.3 Bioanalytical methods

Solid‐phase extraction was used to extract radiprodil from the plasma collected using Aqua‐Cap™ Drummond tubes and conventional sampling, all with a sample volume of 10 μL. Following extraction, all samples were analyzed using a liquid chromatography‐electrospray ionization‐tandem mass spectrometry method. Separation from metabolites and interfering endogenous compounds was achieved by ultra‐performance liquid chromatography using an Acquity HSS T3 column (50 × 2.1 mm, 1.8 μm particles; Waters, Hertfordshire, UK) at 40°C and gradient elution using 0.1% formic acid in water as mobile phase A and acetonitrile as mobile phase B, operating at a flow rate of 0.650 mL min^−1^. The lower limit of quantification (LLOQ) for radiprodil was 1 ng mL^−1^ and the quantification range was 1‐1000 ng mL^−1^. Back‐calculated calibration standard concentrations and quality control sample concentrations for radiprodil in plasma samples are shown in Tables S1 and S2.

### 2.4 Pharmacokinetics

Standard PK parameters were determined from the plasma radiprodil concentrations by noncompartmental analysis. PK parameters were: area under the plasma concentration versus time curve from time 0 to infinity (AUC_inf_); area under the plasma concentration versus time curve from time 0 to the last quantifiable data point (AUC_(0‐t)_); maximum plasma concentration (*C*_max_); terminal half‐life (*t*_&frac12;_); terminal elimination rate constant (*λ*_*z*_); apparent total body clearance (CL/*F*); apparent volume of distribution (*V*_*z*_/*F*); and time to maximum plasma concentration (*t*_max_).

The linear trapezoidal method was used for calculation of AUC values. AUC_inf_ was calculated as AUC_(0‐t)_ + *C*_*t*_/*λ*_*z*_, where *C*_*t*_ is the last observed quantifiable concentration, and *λ*_*z*_ is the slope of linear regression of the natural logarithm (ln) of concentration versus time during the terminal phase of the plasma concentration versus time profile. Apparent terminal *t*_&frac12;_ was calculated as ln(2)/*λ*_*z*_, CL/*F* was calculated as dose/AUC_inf_, and *V*_*z*_/*F* was calculated as (CL/*F*)/*λ*_*z*_. The analysis was performed using WinNonlin^®^ version 6.3 (Certara Inc, Princeton, NJ).

### 2.5 Mitra™ and Aqua‐Cap™ Drummond microsampling

**Figure 1:**
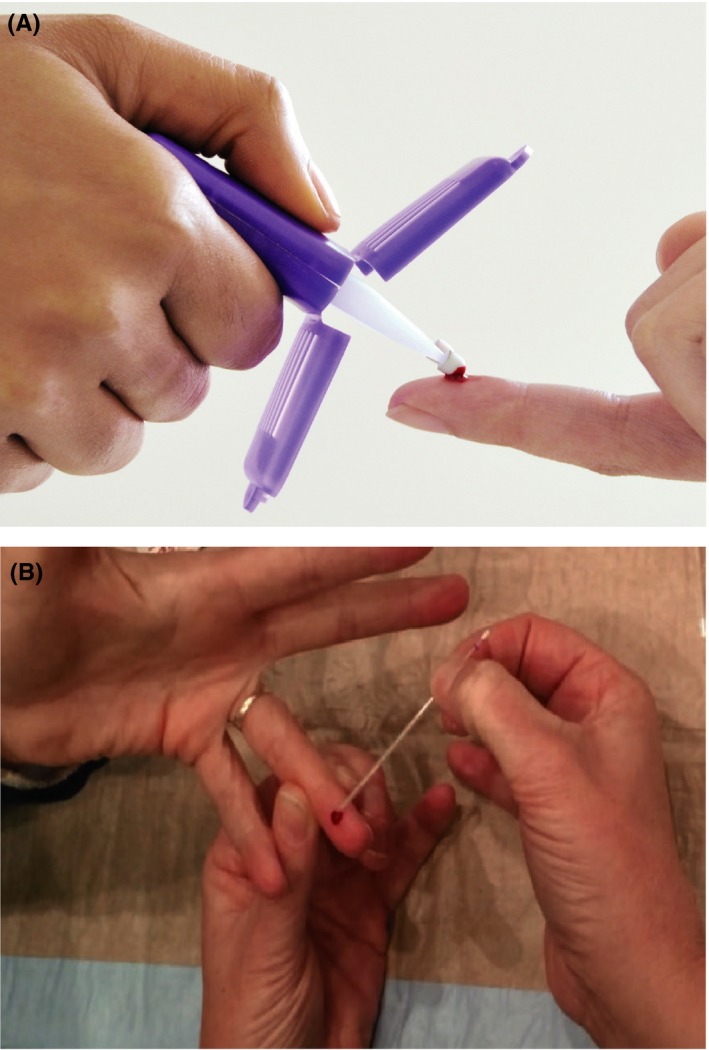
Microsampling techniques: (A) Mitra™ technique and (B) Aqua‐Cap™ Drummond technique

Finger‐prick (peripheral) blood samples were taken from the participants at the same time as the conventional venous samples using two microsampling techniques. The Mitra™ volumetric absorbent microsamplers (Neoteryx, Torrance, CA, https://www.neoteryx.com/; Figure 1A) were dipped into the finger‐prick blood spot, allowing 10 μL of blood to be adsorbed into the tip in 4‐6 seconds. The tips were then allowed to air‐dry for a minimum of 2 hours at 21°C,^2^ allowing for subsequent assay of radiprodil in the whole blood matrix.

Aqua‐Cap™ Drummond capillary tubes (Aqua‐Cap™ Drummond Scientific Company, Philadelphia, PA, https://www.drummondsci.com/; Figure 1B) are specially designed microcapillary separation tubes that were used to draw up 70 μL of whole blood from the finger prick, and were then centrifuged to obtain the plasma for measurement of radiprodil.^3^ Additional (venous) microsamples, using both Mitra™ absorbent microsamplers (10 μL) and Aqua‐Cap™ Drummond tubes (70 μL), were taken from the conventional venous blood sample tubes, prior to centrifugation of the venous samples, in order to assess any differences between venous and peripheral collection using the microsampling techniques. The Mitra™ samples were taken at every scheduled PK sample time point for venous sampling, whereas the Aqua‐Cap™ Drummond samples were only taken at the following time points: 2.5, 3 and 3.5 hours (the expected *C*_max_ for radiprodil).

### 2.6 Microsampling bioanalytical methods

Highly sensitive bioanalytical methods were established and validated in line with internationally recognized standards.^4^ Two independent methods were validated for the measurements of radiprodil in plasma harvested from blood collection using Aqua‑Cap™ Drummond sampling and conventional techniques, and whole blood was collected using Mitra™ sampling.

Radiprodil was extracted from the Mitra™‐derived blood samples using organic extraction, and from the Aqua‐Cap™ Drummond tubes‐derived plasma samples using solid‐phase extraction, all with a sample volume of 10 μL. Following extraction, all samples were analyzed using a liquid chromatography‐electrospray ionization‐tandem mass spectrometry method, as explained above. Back‐calculated calibration standard concentrations and quality control sample concentrations for radiprodil in plasma and dried blood samples are shown in Tables S1‐S4.

### 2.7 Statistical analysis

No formal sample size calculation was performed, due to the exploratory nature of the study. Geometric least squares means (LSMs) and 95% confidence intervals (CI) were calculated for AUC_inf_, AUC_(0‐t)_ and *C*_max_, after logarithmic transformation. Bioequivalence of the microsampling methods to the conventional venous sampling method was tested from the ratio (microsampling versus conventional venous sampling) of back‐transformed geometric LSMs for AUC_inf_, AUC_(0‐t)_,and *C*_max_. Samples collected using different sampling methods were considered bioequivalent if the 90% CIs fell within the bioequivalence range of 0.80‐1.25.

As an additional analysis, Bland‐Altman plots were used to graphically display the level of agreement between plasma concentrations obtained using microsampling versus those obtained using conventional venous sampling. Presenting the difference this way enabled an average discrepancy or bias to be estimated and confirmed if it was constant across the range of values observed. Bland‐Altman plots were developed by obtaining the mean of the two measures (conventional venous and microsampling) for each participant and plotting the mean against the difference of the microsampling value from the conventional venous value for the participant. These plots were built using log‐transformed data. Corresponding Bland‐Altman percent agreement estimates were computed and the Bland‐Altman limits of agreement (95% CI) were calculated for each plot. Statistical calculations were performed using SAS^®^ version 9.4 (SAS Institute, Cary, NC).

## 3 RESULTS

Ten healthy adult participants were enrolled and all completed the study. Seven participants were female, the mean age was 24.3 years (range 19‐45 years), the mean body weight was 73.1 kg (range 51.8‐104.2 kg), and the mean body mass index was 23.6 kg m^−^² (range 18.6‐28.0 kg m^−^²). Nine of the ten participants were white, one was black.

All participants received a single dose of radiprodil and were included in the full analysis set and the PK per‐protocol set (PK‐PPS). There were no major protocol deviations.

### 3.1 Pharmacokinetics

**Figure 2:**
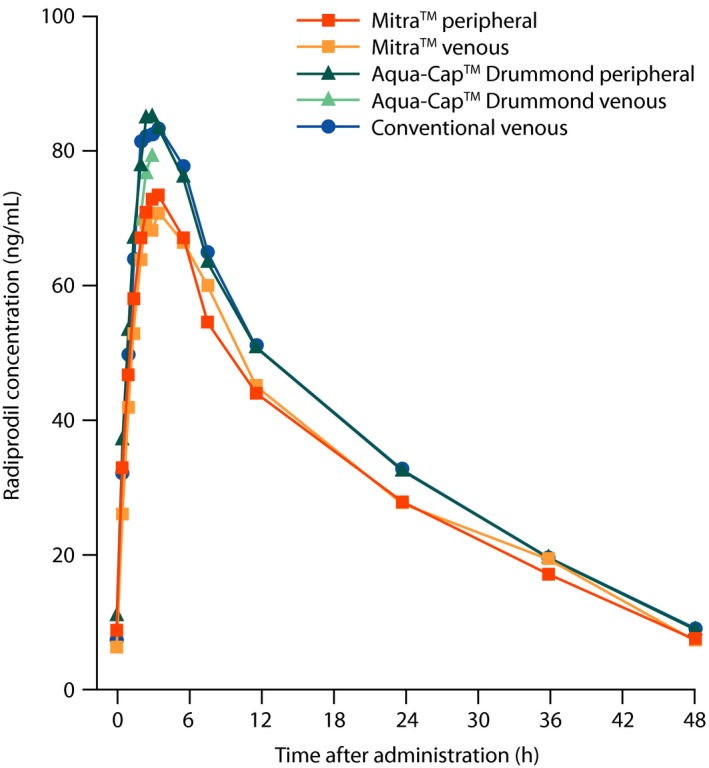
Geometric mean of radiprodil plasma concentration versus scheduled time following administration of radiprodil 30 mg oral suspension (PK‐PPS): comparison of Mitra™ and Aqua‐Cap™ Drummond microsampling techniques with conventional sampling on linear scale. *Note:* All sampling techniques were carried out on all participants (N =  10), at every time point described in the methods. This was with exception of Aqua‐Cap™ Drummond (venous) samples which were only calculated for three time points (2.5, 3 and 3.5 hours). PK‐PPS, pharmacokinetic per protocol set

The primary objective of this study was to evaluate the PK of radiprodil oral suspension in healthy adults. Geometric mean radiprodil plasma concentrations from conventional venous samples reached a maximum at approximately 4 hours, and began to decrease at ~6 hours, approaching the LLOQ 48 hours after administration (Figure 2). Radiprodil oral suspension PK parameters from conventional venous samples are summarized in Table 1. The geometric mean of AUC_inf_, AUC_(0‐t)_, and *C*_max_ following a single dose of radiprodil 30 mg oral suspension was 2042 h ng mL^−1^, 1772 h ng mL^−1^, and 89.4 ng mL^−1^, respectively. The geometric mean *t*_&frac12;_ was 15.8 hours and the median *t*_max_ (range) was 4 hours (3‐6 hours) (Table 1). Inter‐individual variability in the rate and extent of systemic exposure was generally low for conventional sampling at a radiprodil dose of 30 mg. The geometric coefficient of variation (geoCV%) was ≤27.1% for AUC_inf_ and ≤28.6% for *C*_max_.

**Table 1:** Pharmacokinetic parameters from conventional venous samples and Mitra™ and Aqua‐Cap™ Drummond microsamples following administration of radiprodil 30 mg oral suspension (PK‐PPS)

Parameter	Conventional venous blood sampling (n = 10)	Mitra™		Aqua‐Cap™ Drummond^a^
		Peripheral n = 10	Venous n = 10	Peripheral n = 10
Geometric mean (geometric CV [%])				
AUC_inf_ (h ng mL^−1^)	2042 (27.1)	1749 (29.0)	1778 (27.7)	2050 (28.1)
AUC_(0‐t)_ (h ng mL^−1^)	1772 (23.7)	1531 (26.4)	1565 (25.4)	1777 (24.0)
*C*_max_ (ng mL^−1^)	89.4 (28.6)	80.0 (35.8)	77.6 (32.3)	91.9 (30.3)
*t*_&frac12;_ (h)	15.8 (37.3)	15.5 (34.2)	15.5 (35.6)	15.7 (39.2)
*λ*_*z*_ (h^−1^)	0.0439 (37.3)	0.0448 (34.2)	0.0447 (35.6)	0.0440 (39.2)
CL/*F* (L)	14.7 (27.1)	17.2 (29.0)	16.9 (27.7)	14.6 (28.1)
*V*_*z*_/*F* (L)	334 (32.5)	383 (34.2)	378 (34.3)	332 (32.1)
Median (min, max)				
*t*_max_ (h)	4.0 (3.0, 6.0)	4.0 (3.0, 8.0)	3.8 (3.0, 8.0)	3.8 (3.0, 8.0)

AUC_inf_, area under serum concentration‐time curve from time 0 to infinity; AUC_(0‐t)_, area under plasma concentration‐time curve from time 0 to the last quantifiable time point; CL/*F*, apparent total body clearance; *C*_max_, observed maximum concentration; CV, coefficient of variation; max, maximum; min, minimum; PK‐PPS, pharmacokinetic per‑protocol set; *t*_&frac12;_, terminal half‐life; *t*_max_, time to *C*_max_; *V*_z_/*F*, apparent volume of distribution; *λ*_*z*_, terminal elimination rate constant.

Aqua‐Cap™ Drummond venous sampling was carried out at only three time points (2.5, 3 and 3.5 hours) and, therefore, no parameters were calculated.

### 3.2 Safety and tolerability

The secondary objectives of this study were to evaluate the safety and tolerability of radiprodil oral suspension. During the study, 12 treatment‐emergent adverse events (TEAEs) were reported by eight of the ten participants. Of these, ten TEAEs in eight participants were considered to be drug related. The most frequently reported TEAEs were dizziness (4/10 participants; 40%), nausea (2/10; 20%), and somnolence (2/10; 20%). All TEAEs were mild in severity and resolved by the end of the study. There were no severe TEAEs, no serious TEAEs, no discontinuations due to TEAEs, and no deaths. During the study there were no clinically relevant changes in hematology, biochemistry, urinalysis, or vital signs, and no clinically relevant electrocardiogram abnormalities.

### 3.3 Palatability

An exploratory objective of this study was to evaluate the palatability of radiprodil oral suspension. All participants found radiprodil acceptable to swallow, with an acceptable taste, and nine of ten subjects found the texture of the formulation acceptable.

### 3.4 Comparison of conventional and microsampling techniques

Two separate but simultaneous PK sample data sets were acquired for each of the two microsampling techniques (Mitra™ and Aqua‐Cap™ Drummond), one from blood taken directly from a finger prick (peripheral; Figure 1) and one from the blood sample taken using the conventional venous method. PK parameters were not calculated for the Aqua‐Cap™ Drummond venous samples, as these were only collected at 2.5, 3, and 3.5 hours.

As with the conventional venous samples, radiprodil *C*_max_ for the microsampling techniques was reached at approximately 4 hours and approached the LLOQ 48 hours after administration. Marginally lower radiprodil concentrations over the treatment period were observed using Mitra™ (venous and peripheral) sampling compared with Aqua‐Cap™ Drummond (venous and peripheral) and conventional venous sampling (Figure 2).

The geometric mean values of AUC_inf_, AUC_(0‐t)_, and *C*_max_ were similar for the Aqua‐Cap™ Drummond peripheral sampling group compared with the conventional venous group. However, these values were lower for both of the Mitra™ sampling groups (peripheral and venous) compared with the conventional venous group (Table 1). The geometric mean values of the remaining PK parameters were similar across all sampling groups. The AUC_inf_, AUC_(0‐t)_, and *C*_max_ parameters derived from Aqua‐Cap™ Drummond and Mitra™ samples were bioequivalent to those of the conventional venous method since the 90% CIs of the geometric mean ratios were within the bioequivalence range of 0.80‐1.25 (Table 2).

**Table 2:** Pharmacokinetic parameters following administration of radiprodil 30 mg oral suspension: comparison of Mitra™ and Aqua‐Cap™ Drummond microsampling techniques with conventional venous sampling (PK‐PPS)

Parameter	Mitra™		Aqua‐Cap™ Drummond^a^
	Peripheral n = 10	Venous n = 10	Peripheral n = 10
Ratio of geometric means^&ddagger;^ (geometric 90% CI)			
AUC_inf_ (h ng mL^−1^)	0.86 (0.83, 0.89)	0.87 (0.84, 0.90)	1.00 (0.97, 1.04)
AUC_(0‐t)_ (h ng mL^−1^)	0.86 (0.83, 0.90)	0.88 (0.85, 0.92)	1.00 (0.97, 1.04)
*C*_max_ (ng mL^−1^)	0.89 (0.85, 0.94)	0.87 (0.82, 0.92)	1.03 (0.97, 1.08)

AUC_inf_, area under serum concentration‐time curve from time 0 to infinity; AUC_(0‐t)_, area under plasma concentration‐time curve from time 0 to the last quantifiable time point; CI, confidence interval; *C*_max_, observed maximum concentration; PK‐PPS, pharmacokinetic per protocol set; *t*_max_, time to *C*_max._

a Aqua‐Cap™ Drummond venous sampling was carried out at only three time points (2.5, 3 and 3.5 hours) and therefore no parameters were calculated. ^&ddagger;^Microsampling versus conventional venous sampling.

Among the PK parameters, the between‐participant variability for AUC_inf_ was similar for the Mitra™ and Aqua‐Cap™ Drummond techniques. For *C*_max_, the between‐participant variability appeared to be a little higher with the microsampling techniques (geoCV% = 30.3%‐35.8%) compared with conventional venous sampling (geoCV% = 28.6%) (Table 1).

**Figure 3:**
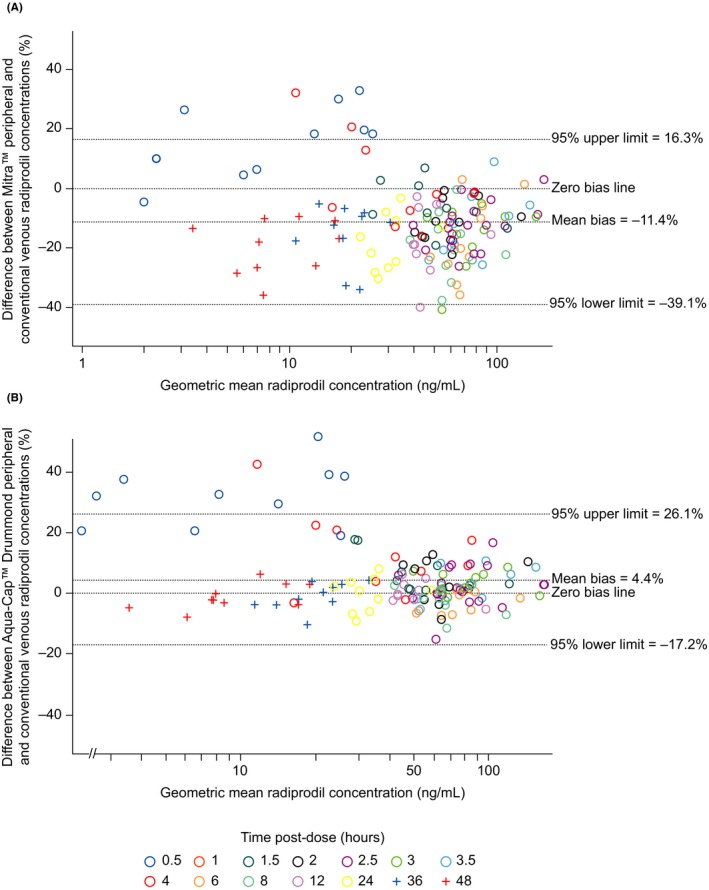
Bland‐Altman plots for radiprodil concentrations (ng mL ^−1^) measured from (A) Mitra™ peripheral and (B) Aqua‐Cap™ Drummond peripheral microsamples compared with conventional venous samples. The *X* axis shows the geometric mean of the radiprodil concentration measured by the two methods (peripheral microsampling and conventional venous). The *Y* axis shows the percentage difference between the radiprodil concentration measured by microsampling and conventional venous sampling (after back‐transformation of log data). *Note:* Accurate prediction with 10% homogeneous error. All sampling techniques were carried out on all patients (N = 10), at every time point described in the methods

The Bland‐Altman comparison of plasma concentrations yielded a mean bias (as measured by geometric mean difference; 95% CI) of −11.4% (−39.1, 16.3) and 4.4% (−17.2, 26.1) for Mitra™ (peripheral) and Aqua‐Cap™ Drummond (peripheral) microsampling, respectively, versus conventional venous sampling. The Bland‐Altman plots confirmed the high association for Aqua‐Cap™ Drummond microsampling with conventional sampling concentrations and also good association for Mitra™ microsampling with conventional sampling concentrations (negative bias less than 12%) (Figure 3).

## 4 DISCUSSION

Clinical studies in infants frequently require an oral liquid suspension or solution formulation of the drug to be developed specifically for this patient population, in line with clinical practice. A major benefit of an oral liquid is that it can be extemporaneously prepared with a diluent or vehicle, and therefore can cover a broad range of doses. This provides flexibility for weight adjustment in the infant setting, where weight changes are rapidly compared with older children. An oral suspension formulation of radiprodil has therefore been developed with this goal in mind.

In this study, carried out in ten healthy adult volunteers, the radiprodil oral suspension formulation had an acceptable safety profile and TEAEs were all mild in severity. The taste and texture of the oral suspension were considered to be acceptable by 10/10 and 9/10 participants, respectively; this is a crucial property for oral liquids because palatability is a major determinant of treatment compliance in infants and children.^5^, ^6^, ^7^ Further confirmatory studies in children of various age groups would be of value given that younger children often demonstrate different palatability preferences from older children and adults.^8^

Marginally lower concentrations of radiprodil were observed over the treatment period using peripheral and venous Mitra™ microsampling compared with Aqua‐Cap™ Drummond (peripheral and venous) microsampling and conventional venous sampling. This may be a reflection of the blood to plasma partition of radiprodil, and could be explained by the different matrices tested: plasma from samples collected using the Aqua‐Cap™ Drummond capillary tubes and conventional sampling, and whole blood samples collected using Mitra™ volumetric absorbent samplers. However, despite these lower concentrations, the PK profile of radiprodil from samples collected via the Mitra™ and Aqua‐Cap™ Drummond microsampling methods demonstrated bioequivalence in terms of 90% CIs of the geometric mean ratio to that obtained using the conventional venous collection techniques, regardless of the site of microsample collection. This confirms that either technique is fit for purpose for use in future clinical studies with radiprodil. These less invasive microsampling techniques have great potential to facilitate and add value to any paediatric programme. They are particularly beneficial in this population because they may allow a PK profile to be measured without the need for a venous line, and with a sample volume of no more than 70 μL; in particular, Mitra™ microsampling only requires 10 μL of blood. In the case of Mitra™ volumetric absorbent samplers, the microsampling technique obviates the need for centrifugation, plasma separation, and freezing (including frozen shipment), thus reducing the processing burden of blood sampling. Furthermore, during the prestudy bioanalytical method validation the impact of hematocrit was assessed and no effect was found across a supra‐ and infra‐normal range of hematocrit levels. The phenomenon observed with dried blood spot samples (DBS), where samples spread across a DBS card in a heterogeneous manner due to variability in hematocrit, and the subsequent impact of taking a sub‐section of the blood spot for analysis, has largely been resolved by Mitra™, since (unlike DBS), Mitra™ collects a fixed (10 μL) volume of blood with extremely low levels of variability (<5%). This technique allows the potential for collection of samples at home or in remote areas where access to a clinic may be challenging. The key limitation of the study's relevance to infants is that it was conducted in adults. There is, however, no a priori reason why either microsampling technique should not work just as well in the infant setting.

In conclusion, the PK profile of radiprodil single‐dose 30 mg oral suspension was established in healthy adults, and was comparable when analyzed using conventional versus microsampling techniques. Radiprodil had acceptable safety and palatability profiles in this population. Results from this study will therefore inform and facilitate future paediatric radiprodil studies. A study to evaluate the safety, tolerability, PK, and efficacy of radiprodil in infants 2‐14 months of age with drug‑resistant IS is currently ongoing (NCT02829827).

## CONFLICTS OF INTEREST

Jan Jaap van Lier is an employee of PRA Health Services. David Sciberras, Christian Otoul, Françoise Lurquin, John Smeraglia, Aurélia Lappert, and Steven De Bruyn are employees of UCB Pharma.
