# Supplementary material for: A pharmacokinetic study of radiprodil oral suspension in healthy adults comparing conventional venous blood sampling with two microsampling techniques
Source: Pharmacol Res Perspect. 2019 Jan 28;7(1):e00459. doi: 10.1002/prp2.459 (PMC6349788; doi:10.1002/prp2.459)
Supplement: Supplementary file 1 [file PRP2-7-e00459-s001.docx]

**A pharmacokinetic study of radiprodil oral suspension in healthy adults comparing conventional venous blood sampling with two microsampling techniques**

David Sciberras^1^, Christian Otoul^1^, Françoise Lurquin^1^, John Smeraglia^1^, Aurélia Lappert^1^, Steven De Bruyn^1^, Jan Jaap van Lier^2^

Table S1 Back-calculated calibration standard concentrations for radiprodil in plasma samples

|  | **Calibration sample concentrations (ng mL^-1^)** | | | | | | | | |
| --- | --- | --- | --- | --- | --- | --- | --- | --- | --- |
| **Run** | **1.00** | **2.00** | **5.00** | **20.0** | **50.0** | **200** | **500** | **800** | **1000** |
| 1 | 1.02 | 1.92 | 4.96 | 19.9 | 50.2 | 208 | 505 | 828 | 943 |
| 2 | 0.99 | 2.07 | 4.85 | 20.1 | 49.2 | 203 | 510 | 800 | 979 |
| 3 | 1.02 | 1.92 | 4.84 | 20.7 | 51.1 | 202 | 510 | 823 | 932 |
| 4 | 1.02 | 1.93 | 5.04 | 20.2 | 51.9 | 197 | 507 | 792 | 978 |
| **Overall statistics** |  |  |  |  |  |  |  |  |  |
| Mean (ng mL^-1^) | 1.01 | 1.96 | 4.92 | 20.2 | 50.6 | 203.0 | 508.0 | 811.0 | 958.0 |
| CV (%) | 1.60 | 3.80 | 1.90 | 1.7 | 2.3 | 2.2 | 0.5 | 2.2 | 2.5 |
| Bias (%) | 1.00 | -2.00 | -1.60 | 1.0 | 1.2 | 1.5 | 1.6 | 1.4 | -4.2 |
| n | 4 | 4 | 4 | 4 | 4 | 4 | 4 | 4 | 4 |

Calculations were done with rounded values.

CV, coefficient of variation

Table S2 Quality control sample concentrations for radiprodil in plasma samples

|  | **Quality control sample concentrations (ng mL^-1^)** | | | | | | | |
| --- | --- | --- | --- | --- | --- | --- | --- | --- |
| **Run** | **3.00** | |  | **50.0** | |  | **800** | |
|  | **Concentration (ng mL^-1^)** | **Bias (%)** |  | **Concentration (ng mL^-1^)** | **Bias (%)** |  | **Concentration (ng mL^-1^)** | **Bias (%)** |
| 1 | 3.05 | 1.7 |  | 52.1 | 4.2 |  | 845 | 5.6 |
|  | 3.22 | 7.3 |  | 51.6 | 3.2 |  | 820 | 2.5 |
| 2 | 3.04 | 1.3 |  | 48.3 | -3.4 |  | 806 | 0.8 |
|  | 2.97 | -1.0 |  | 47.7 | -4.6 |  | 782 | -2.3 |
| 3 | 3.18 | 6.0 |  | 51.4 | 2.8 |  | 798 | -0.3 |
|  | 2.86 | -4.7 |  | 48.9 | -2.2 |  | 818 | 2.3 |
| 4 | 3.08 | 2.7 |  | 47.7 | -4.6 |  | 803 | 0.4 |
|  | 2.87 | -4.3 |  | 51.2 | 2.4 |  | 792 | -1.0 |
| **Overall statistics** |  |  |  |  |  |  |  |  |
| Mean (ng mL^-1^) | 3.03 |  |  | 49.9 |  |  | 808 |  |
| CV (%) | 4.3 |  |  | 3.8 |  |  | 2.4 |  |
| Bias (%) | 1.0 |  |  | -0.2 |  |  | 1.0 |  |
| n | 8 |  |  | 8 |  |  | 8 |  |

Calculations were done with rounded values.

CV, coefficient of variation

Table S3 Back-calculated calibration standard concentrations for radiprodil in dried blood samples

|  | **Calibration sample concentrations (ng mL^-1^)** | | | | | | | | |
| --- | --- | --- | --- | --- | --- | --- | --- | --- | --- |
| **Run** | **1.00** | **2.00** | **5.00** | **20.0** | **50.0** | **200** | **500** | **800** | **1000** |
| 1 | 1.00 | 1.99 | 4.99 | 20.8 | 49.2 | 202 | 523 | 731 | 1010 |
| 2 | 0.98 | 2.03 | 5.22 | 21.7 | 48.9 | 191 | 537 | 747 | 938 |
| 3 | 0.96 | 2.10 | 5.39 | 19.5 | 48.4 | 200 | 483 | 797 | 1010 |
| **Overall statistics** |  |  |  |  |  |  |  |  |  |
| Mean (ng mL^-1^) | 0.98 | 2.04 | 5.20 | 20.7 | 48.8 | 198 | 514 | 758 | 986 |
| CV (%) | 2.0 | 2.7 | 3.9 | 5.4 | 0.8 | 3.0 | 5.4 | 4.5 | 4.2 |
| Bias (%) | -1.9 | 2.0 | 4.0 | 3.5 | -2.4 | -1.0 | 2.8 | -5.3 | -1.4 |
| n | 3 | 3 | 3 | 3 | 3 | 3 | 3 | 3 | 3 |

Calculations were done with rounded values.

CV, coefficient of variation

Table S4 Quality control sample concentrations for radiprodil in dried blood samples

|  | **Quality control sample concentrations (ng mL^-1^)** | | | | | | | |
| --- | --- | --- | --- | --- | --- | --- | --- | --- |
| **Run** | **3.00** | |  | **50.0** | |  | **800** | |
|  | **Concentration (ng mL^-1^)** | **Bias (%)** |  | **Concentration (ng mL^-1^)** | **Bias (%)** |  | **Concentration (ng mL^-1^)** | **Bias (%)** |
| 1 | 3.08 | 2.7 |  | 54.3 | 8.6 |  | 749 | -6.4 |
|  | 3.01 | 0.3 |  | 57.3 | 14.6 |  | 875 | 9.4 |
| 2 | 3.78 | 26.0^†^ |  | 52.2 | 4.4 |  | 759 | -5.1 |
|  | 3.09 | 3.0 |  | 57.2 | 14.4 |  | 820 | 2.5 |
| 3 | 3.43 | 14.3 |  | 57.2 | 14.4 |  | 783 | -2.1 |
|  | 2.88 | -4.0 |  | 53.7 | 7.4 |  | 888 | 11.0 |
| **Overall statistics** |  |  |  |  |  |  |  |  |
| Mean (ng mL^-1^) | 3.21 |  |  | 55.3 |  |  | 812 |  |
| CV (%) | 10.4 |  |  | 4.0 |  |  | 7.3 |  |
| Bias (%) | 7.0 |  |  | 10.6 |  |  | 1.5 |  |
| n | 6 |  |  | 6 |  |  | 6 |  |

Calculations were done with rounded values.

^†^Value outside the QC limits.

CV, coefficient of variation
